# Combined online and offline basic life support workshop with infection prevention and control for COVID‐19

**DOI:** 10.1002/jgf2.538

**Published:** 2022-04-19

**Authors:** Akihiro Ikeda, Yoshihiro Tochino, Tomoya Nishihata, Sachiko Oku, Taichi Shuto

**Affiliations:** ^1^ Osaka City University School of Medicine Osaka Japan; ^2^ Department of Medical Education and General Practice, Osaka City University Graduate School of Medicine Osaka Japan; ^3^ Skills Simulation Center Osaka City University Hospital Osaka Japan

## Abstract

The coronavirus disease 2019 outbreak has made it difficult to hold face‐to‐face BLS training sessions at university. Even in this limited situation, the effective use of combined online video course and offline training can contribute to gaining participants’ confidence in conducting BLS and improving mindset than before.

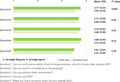

To the Editor,

Recovery after out‐of‐hospital cardiac arrest (OHCA) is still one of the most important public health concerns around the world^1^, ^2^. Immediate recognition and delivery of high‐quality basic life support (BLS) is one of the most critical factors in survival from OHCA^3^. Survival rates after bystander‐witnessed shockable OHCA widely ranged from 11.7% to 47.4%^1^. Based on this evidence, the “Life Support Club (LSC)” was organized at Osaka City University School of Medicine to enable people in the community and medical students to learn BLS. Since then, we have conducted BLS training sessions more than twenty times a year.

However, the coronavirus disease 2019 outbreak has made it difficult to hold face‐to‐face training sessions at university^4^. Even under such circumstances, the levels of willingness to provide cardiopulmonary resuscitation have not decreased^5^. To resolve this difficult situation, we tried to provide a combined online‐and‐offline‐based BLS workshop.

The participants were first‐year medical students at Osaka City University School of Medicine, and the instructors were fourth‐year medical students. We used Resusci® Anne manikin for training. In previous years, two instructors and two students were assigned to each booth, but this year, we decided to assign only one instructor and one student to avoid overcrowding. The workshop was implemented with slides and videos, followed by repeated practical exercises. For infection control, hand cleaning, wearing of masks, disinfection of Resusci® Anne manikin, and keeping the distance between booths were taken. In addition, all participants and instructors were given a video about BLS and a confirmation test we created prior to the seminar, which reduced the time of training session from 60 min to 45 min, and a questionnaire was administered after the workshop.

92 participants (92/95, 96.8%) responded to the questionnaire survey after attending the course this year, compared with the previous year of 68 participants (68/95, 71.6%). A 4‐point Likert scale (1 = strongly disagree and 4 = strongly agree) was used in the questionnaire. Differences in participants' scores for each question between this year and the previous year were assessed using the Mann–Whitney *U* test. The scores were 3.77 ± 0.42 for “Can you verify scene safety, check for responsiveness, shout for nearby help, and get automated external defibrillator (AED)?”; 3.71 ± 0.46 for “Can you look for no breathing or only gasping?”; 3.76 ± 0.45 for “Can you perform chest compression?”; 3.80 ± 0.40 for “Can you use AED?”; and 3.51 ± 0.52 for “When you found someone down, can you attempt BLS?” (Figure 1). Compared with the results of the previous workshop, those of the first four questions indicate that the participants were more confident in conducting BLS and that of the last one shows that their mindset was enhanced, while not all results are statistically significant.

**Figure 1:**
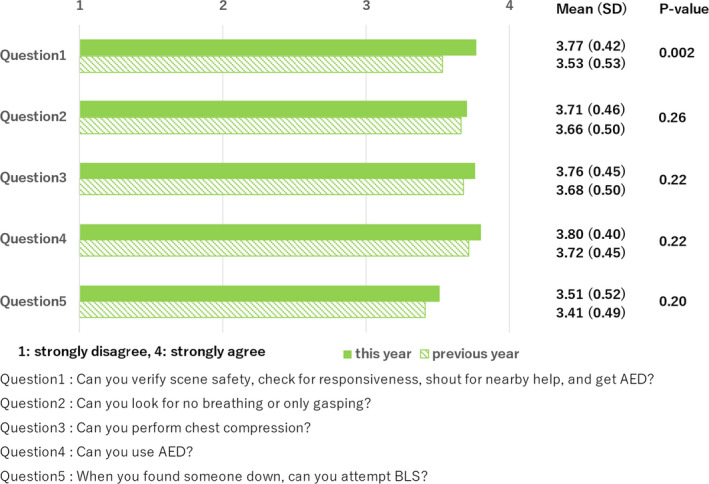
Results of the questionnaire survey both this year and the previous year

A limitation of this study is that we were unable to measure the participants' improvement in BLS skills objectively. In particular, the appropriate standard for chest compressions has been established and we may employ a device to measure them.

Even in this limited situation, the effective use of blended online video course and offline training can contribute to gaining participants' confidence in conducting BLS and improving mindset than before. LSC will strive to teach medical students and people in the community how to carry out BLS for the years to come and hopes that it will lead to saving as much life as possible.

## CONFLICT OF INTEREST

None.
